# Selective inhibition of histone deacetylase 8 improves vascular hypertrophy, relaxation, and inflammation in angiotensin II hypertensive mice

**DOI:** 10.1186/s40885-019-0118-8

**Published:** 2019-06-15

**Authors:** Hae Jin Kee, Yuhee Ryu, Young Mi Seok, Sin Young Choi, Simei Sun, Gwi Ran Kim, Myung Ho Jeong

**Affiliations:** 1 Heart Research Center of Chonnam National, Jebong-ro, Dong-gu, Gwangju, 61469 Republic of Korea; 2 Hypertension Heart Failure Research Center, Chonnam National University Hospital, Gwangju, 61469 Republic of Korea; 3 National Development Institute of Korean Medicine, Hwarang-ro, Gyeongsan-si, Gyeongsangbuk-do Republic of Korea; 4 Molecular Medicine, Brain Korea 21 PLUS, Chonnam National University Graduate School, Gwangju, 61469 Republic of Korea

**Keywords:** PCI34051, Hypertension, Arterial remodeling, Vascular relaxation, Inflammation

## Abstract

**Background:**

The dysregulation of histone deacetylase (HDAC) protein expression or its enzyme activity is implicated in a variety of diseases. Cardiac HDAC6 and HDAC8 enzyme activity induced by deoxycorticosterone acetate (DOCA) hypertension was attenuated by sodium valproate, a pan-HDAC inhibitor. However, the HDAC6-selective inhibitor, tubastatin A, did not attenuate angiotensin II-induced hypertension. The purpose of this study was to investigate whether PCI34051, an HDAC8-selective inhibitor, can modulate angiotensin II-induced hypertension and its regulatory mechanism.

**Methods:**

An angiotensin II-regulated mouse model was used in this study. Animals received vehicle or PCI34051 (3 mg·kg − ^1^·day− ^1^) via intraperitoneal injection. Systolic blood pressure was measured by the tail-cuff method. Blood vessel thickness was measured following hematoxylin and eosin staining, VCAM-1 immunohistochemistry was performed in the aortas, and mRNA expression of renin-angiotensin system components, inflammation markers, and NADPH oxidase (Nox) was determined by RT-PCR. The effect of PCI34051 on vasorelaxation was studied in rat aortic rings, and its effect on nitric oxide (NO) production was determined using DAF-FM DA, a fluorescent dye, in human umbilical vascular endothelial cells (HUVECs).

**Results:**

PCI34051 administration reduced systolic blood pressure via downregulation of angiotensin II receptor type 1 (AT1) mRNA expression. PCI34051 treatment attenuated vascular hypertrophy by decreasing E2F3 and GATA6 mRNA expression. Vascular relaxation after PCI34051 treatment was more dependent on vascular endothelial cells and it was blocked by an NO synthase (NOS) inhibitor. In addition, NO production increased in HUVECs after PCI34051 treatment; this was decreased by the NOS inhibitor. The expression of inflammatory molecules and adhesion molecules VCAM-1 and ICAM-1 decreased in the aortas of angiotensin II-infused mice after PCI34051 administration. However, PCI34051 did not affect Nox or its regulatory subunits.

**Conclusions:**

PCI34051 lowered high blood pressure through modulation of arterial remodeling, vasoconstriction, and inflammation in an angiotensin II-induced hypertension model. We suggest that HDAC8 could be a potential therapeutic target for hypertension.

## Background

Continuously high blood pressure causes multiple complications, including stroke, hypertensive retinopathy, myocardial infarction, heart failure, and chronic renal failure [1, 2]. High blood pressure is known as a silent killer because there are no symptoms and therefore it can go untreated for long periods of time. It is known that the control rate is no more than 40% even when hypertensive patients receive two or more antihypertensive drugs [3]. Inflammatory responses are involved in the pathophysiology of hypertension [4]. Recent evidence has revealed inflammation in the vessel structure of animal models of hypertension [5].

Angiotensin II is a vasoconstrictor, causing vascular inflammation in arteries as well as in the kidneys and heart [6, 7]. It increases the expression of adhesion molecules, cytokines, and chemokines in endothelial cells and vascular smooth muscle cells [8]. Vascular cell adhesion molecule-1 (VCAM-1), intercellular adhesion molecule-1 (ICAM-1), and platelet endothelial cell adhesion molecule (PECAM) may be implicated in hypertension [9]. However, soluble E-selectin has been significantly associated with blood pressure [10]. In contrast, soluble VCAM-1 (sVCAM-1) is elevated in older men with uncomplicated essential hypertension [11]. Recent evidence showed that expression of VCAM-1 is positively correlated with coronary lesion severity in atherosclerotic patients [12]. In addition, VCAM-1 was reported to be a biochemical marker of left ventricular mass in patients with uncomplicated hypertension [13].

The renin-angiotensin system (RAS) is the most important and well-known hormone system for controlling hypertension. Angiotensin-converting enzyme (ACE) converts angiotensin I to angiotensin II. Angiotensin II acts through angiotensin II receptor type 1 (AT1), which leads to vasoconstriction and inflammation [14]. An angiotensin II receptor blocker (ARB) reduces high blood pressure and inflammation by interfering with AT1 binding. Currently, ACE inhibitors and ARBs are widely used as hypertension treatments in clinical trials.

Hypertension is caused by increased resistance of blood vessels. Blood vessels are mainly composed of smooth muscle cells and endothelial cells. Endothelial cells secret substances that relax blood vessels, such as nitric oxide (NO), and substances that contract blood vessels, for example, endothelin-1. Vascular smooth muscle cells control blood pressure mainly through vascular contraction and relaxation. Contraction of smooth muscle cells is mainly due to changes in calcium concentration. Phosphorylation of myosin light chains is effected by myosin light chain kinase (MLCK), and arterial smooth muscle contracts through Ca^2+^/MLCK pathways [15, 16].

Histone deacetylases (HDACs) and their inhibitors have been extensively studied in cancer and they are now attracting attention in cardiovascular disease research [17]. HDACs are classified into class I (HDAC1, 2, 3, and 8), class IIa (HDAC4, 5, 7, and 9), class IIb (HDAC6 and 10), class III (sirtuins1-7), and class IV (HDAC11), based on their sequence. HDAC inhibitors have been shown to be effective in cardiac hypertrophy, fibrosis, heart failure, and restenosis [18–20]. Furthermore, the HDAC inhibitor, valproic acid, attenuated blood pressure in spontaneously hypertensive rats and deoxycorticosterone acetate (DOCA)- induced hypertensive rats [21, 22]. However, there was no use of isoform-specific HDAC inhibitors in these reports. We have published a study showing that HDAC6 and 8 enzyme activities are increased in the hearts of DOCA hypertensive rats [23]. We demonstrated that the HDAC6-selective inhibitor, tubastatin A, did not affect high blood pressure in an angiotensin II-induced hypertensive mouse model. Therefore, we hypothesized that HDAC8 may have a role in hypertension. To provide evidence supporting this hypothesis, we decided to investigate the effect of the HDAC8-selective inhibitor, PCI34051, on blood pressure in angiotensin II-induced hypertensive mice.

## Methods

### Animal model and blood pressure measurement

Angiotensin II (Ang II) was obtained from EMD Millipore (Billerica, MA, USA). PCI34051 was purchased from Selleckchem (Houston, TX, USA). CD-1 male mice (aged 8 weeks) were purchased from Orient Bio Company (Gyeonggi-do, South Korea). All animal experiments were approved by the Animal Experiment Committee of the Chonnam National University Medical School (CNU IACUC-H-2017-70) and carried out in accordance with the Guide for the Care and Use of Laboratory Animals (US National Institutes of Health Publication, 8th edition, 2011). Mice were anesthetized with ketamine (120 mg/kg) and xylazine (6.21 mg/kg), and a 1 cm incision was made in the back. Ang II (1.3 mg·kg^−1^·day^−1^, n=8 per group) was infused via an ALZET® osmotic pump as described previously. Ang II was dissolved in 0.9% NaCl solution. The sham control mice received dimethyl sulfoxide vehicle (DMSO; n=8). PCI34051 (3 mg·kg^−1^·day^−1^) was administered to the mice (n=8 per group) by intraperitoneal injection from the eighth to the fourteenth day after Ang II infusion began. Blood pressure was measured three times a week in conscious animals by the tail-cuff method. Systolic blood pressure was determined on the fourteenth day after Ang II infusion began.

### Hematoxylin and eosin (H&E) staining

Aorta tissues were fixed in 4% paraformaldehyde at 25°C, embedded in paraffin, and cut into 3 μm thick sections. The tissue slides were deparaffinized three times with xylene and hydrated using serially diluted ethanol. After dipping in tap water for 2 min, the slides were stained with Gill’s hematoxylin V for 5 min, washed in tap water for 5 min, and in 95% ethanol for 2 min. The slides were stained using Eosin Y for 1 min, dehydrated with ethanol and xylene, and mounted using Canada balsam. The aortic wall thickness was measured using NIS Elements Software (Nikon, Japan).

### Immunohistochemistry

Aorta tissues were fixed in 4% paraformaldehyde at 25°C, embedded in paraffin, and cut into 3-μm-thick sections. After deparaffinization in xylene, the tissues were subjected to antigen retrieval using 10 mM citrate-phosphate buffer (pH 6.0) and incubated in 3% H2O_2_ for 10 min. To remove non-specific binding, an Avidin/Biotin blocking kit (Abcam, USA) was used, and the sections were then blocked with 1% bovine serum albumin in PBS for 10 min. Sections were incubated overnight with mouse monoclonal VCAM-1 antibody (1:50, Santa Cruz) at 4°C and washed in PBS before being incubated with prediluted biotinylated pan- specific universal secondary antibody (R.T.U. VECTASTAIN kit) for 30 min at 25°C. Tissues were washed with PBS, incubated in streptavidin/peroxidase complex for 5 min, and again washed with PBS. The tissues were incubated in DAB (peroxidase substrate kit, SK-4100) for 7 min. Sections were stained with hematoxylin for 30 s and washed and mounted. Images were captured using a fluorescent microscope (Eclipse 80*i*, Nikon, Japan).

### Isometric tension measurement

The vasoconstriction-relaxation study was performed as described previously. Male Sprague- Dawley rats were purchased from Orient Bio (Gyeonggi-do, South Korea). Briefly, thoracic aortas were excised and immersed in ice-cold modified Krebs solution. The aortas were cleaned of all connective tissue, soaked in Krebs-bicarbonate solution, and cut into four ring segments (3.5 mm in length). Each aortic ring was suspended in a water-jacketed organ bath(6 ml) maintained at 37°C and aerated with a mixture of 95% O_2_ and 5% CO_2_. Each ring was connected to an isometric force transducer (Danish Myo Technology, Skejbyparken, Aarhus N, Denmark). Rings were stretched to an optimal resting tension of 2.0 ×*g* or 1.0 ×*g*, which was maintained throughout the experiment. Each ring was equilibrated in the organ bath solution for 90 min before measuring the contractile response after the addition of 50 mM KCl. To determine the effect of PCI34051 on the maintenance of vascular tension in rat endothelium-intact or endothelium-denuded aortic rings, vascular contractions were induced using the thromboxane A2 agonist, U46619 (30 nM, 20 min). When each contraction reached a plateau, increasing concentrations of PCI34051 (0.003 - 3 μM) were added cumulatively to elicit vascular relaxation.

In the second experiment, we investigated the inhibition of the relaxation response by treating endothelium-intact aortic rings with N^G^-nitro-L-arginine methyl ester (L-NAME, 10 and 100 μM) for 30 min. After U46619 treatment, increasing concentrations of PCI34051 (0.003 - 1 μM) were added cumulatively to the aortic rings.

### Cell culture

Human umbilical vein endothelial cells (HUVECs) were obtained from Gibco (Waltham, MA, USA). HUVECs were grown in endothelial cell basal medium (EBM) with an EGM-2 bullet kit (Lonza, Walkersville, MD) and maintained at 37°C under 5% CO_2_. The cells were subcultured when they reached approximately 90% confluency. Cells were used from passages five to seven. The vascular smooth muscle cells (VSMCs) were isolated from rat aortas as described previously [24]. VSMCs were maintained in low glucose with Dulbecco’s modified Eagle medium (DMEM) containing 10% fetal bovine serum. VSMCs were used from passages five to nine.

### Western blot analysis

VSMCs were lysed with RIPA buffer (150 mM NaCl; 1% Triton X-100; 1% sodium deoxycholate; 50 mM Tris-HCl, pH 7.5; 2 mM EDTA; 1 mM PMSF; 1 mM DTT; 1 mM Na3VO4; and 5 mM NaF) containing a protease inhibitor cocktail. The proteins were subjected to 10% SDS-PAGE and transferred on to polyvinylidene difluoride (PVDF) membranes. The membranes were blocked with 5% skim milk in Tris-buffered saline with Tween® 20 (TBST) buffer (20 mM Tris, 200 mM NaCl, and 0.04% Tween® 20) for 1 h at 25 °C. The membranes were incubated overnight at 4 °C with HDAC8 antibody (Santa Cruz) and then incubated with anti-mouse horseradish-peroxidase-conjugated secondary antibody (1:5000) for 1 h at 25 °C. The protein bands were visualized using Immobilon Western detection reagents (EMD Millipore). Bio-ID software was used to quantify the protein expression (Vilber Lourmat, Eberhardzell, Germany).

### DAF-FM imaging of NO

The production of NO was estimated using a NO-sensitive fluorescence probe, 4-amino-5- methylamino-2′,7′-difluorofluorescein diacetate (DAF-FM DA, Cayman). HUVECs were seeded on coverslips in 12-well plates. Cells were serum-starved with EBM for 16 h, and were treated with PCI34051 (1 μM) or L-NAME (250 μM) for 24 h and then incubated with DAF-FM DA (2.5 μM) at 37°C for 30 min. Cells were fixed using 70% ethanol for 45 min, washed three times with PBS, and mounted using Prolong Gold antifade reagent with DAPI (Invitrogen, USA).

### Reverse transcription polymerase chain reaction

Total RNA was isolated with TriZol reagent (Invitrogen Life Technologies, Waltham, MA, USA), and 1 μg of RNA was used for the reverse transcription reaction using TOPscript RT DryMIX (Enzynomics, Daejeon, South Korea). mRNA expression was determined using the SYBR Green PCR kit (Enzynomics, Daejeon, South Korea). All data were normalized to GAPDH expression using the 2^-ΔΔct^ method. The PCR primers used in this study are shown in Table 1.

**Table 1:** Primers for the reverse transcription polymerase chain reaction (RT-PCR)

Gene (mouse or rat)	Primer sequence (5′ to 3′)
*AT1 (mouse)*	F: GGAAACAGCTTGGTGGTGAT R: GGCCGAAGCGATCTTACATA
*ACE1 (mouse)*	F: CAGTGTCTACCCCCAAGCAT R: TTCCATCAAAGACCCTCCAG
*Nox1 (mouse)*	F: AGCCATTGGATCACAACCTC R: TGGATGGGATTTAGCCAAGA
*Nox2 (mouse)*	F: TGTCATTCTGGTGTGGTTGG R: GAACCCCTGAGGAAGGAGAG
*Nox4 (mouse)*	F: CTGGAAGAACCCAAGTTCCA R: ACTGGCCAGGTCTTGCTTTA
*p22phox (mouse)*	F: AAAGAGGAAAAAGGGCTCCA R: CTGCCAGCAGGTAGATCACA
*p47phox (mouse)*	F: ATCCCAACTACGCAGGTGAA R: TATCTCCTCCCCAGCCTTCT
*GATA6 (mouse)*	F: GAGGACCTGTCGGAGAGCCG R: GCAAGTGGTCGAGGCACCCC
*E2F3 (mouse)*	F: ATCCAAAGCTGTACCCTGGA R: TGGGTACTTGCCAAATGGAT
*iNOS (mouse)*	F: CTCACTGGGACAGCACAGAA R: TGGTCAAACTCTTGGGGTTC
*TNF-α (mouse)*	F: AGCCCCCAGTCTGTATCCTT R: CTCCCTTTGCAGAACTCAGG
*IL-1β (mouse)*	F: GCCCATCCTCTGTGACTCAT R: AGGCCACAGGTATTTTGTCG
*MCP-1 (mouse)*	F: AGGTCCCTGTCATGCTTCTG R: TCTGGACCCATTCCTTCTTG
*IL-6 (mouse)*	F: AGTTGCCTTCTTGGGACTGA R: TCCACGATTTCCCAGAGAAC
*Cox-2 (mouse)*	F: ATCCTGAGTGGGGTGATGAG R: GGCAATGCGGTTCTGATACT
*VCAM-1 (mouse)*	F: TCATCCCCACCATTGAAGAT R: TGAGCAGGTCAGGTTCACAG
*ICAM-1 (mouse)*	F: CCTGTTTCCTGCCTCTGAAG R: TTAAGGTCCTCTGCGTCTCC
*GAPDH (mouse)*	F: GCATGGCCTTCCGTGTTCCT R: CCCTGTTGCTGTAGCCGTATTCAT

F, forward; R, reverse

### Fluorogenic HDAC enzyme activities

To evaluate the HDAC enzyme inhibitory activity of PCI34051, we determined the activities of HDAC1, HDAC2, HDAC3, and HDAC8 using enzyme assay kits (BPS Bioscience, San Diego, CA, USA) according to the manufacturer’s protocols. HDAC activities were measured using a fluorometer (Spectra Max GEMINI XPS, Molecular Devices, Sunnyvale, CA, USA) at excitation and emission wavelengths of 350 nm and 460 nm, respectively. To test the inhibitory activity of PCI34051, concentrations of 0.001, 0.003, 0.01, 0.03, 0.1, 0.3, 1, and 3 μM were used. For the half-maximal inhibitory concentration (IC_50_) calculations, every data point was normalized to the vehicle (100% activity). The normalized data were fitted using a Hill nonlinear curve (OriginPro 9.0). The “Find X from Y” function in OriginPro 9.0 was used to determine the IC_50_ values (50% activity).

### Statistical analyses

Statistical analysis was performed using one-way analysis of variance (ANOVA) followed by the Bonferroni post-hoc test to compare the treatment groups (GraphPad Prism, version 5.0; GraphPad Software, La Jolla, CA, USA). Data are presented as the mean ± SEM. A value of *P* < 0.05 was considered statistically significant.

## Results

### HDAC8-selective inhibitor PCI34051 reduces blood pressure through down-regulation of AT1 in Ang II-induced hypertensive mice

**Fig. 1:**
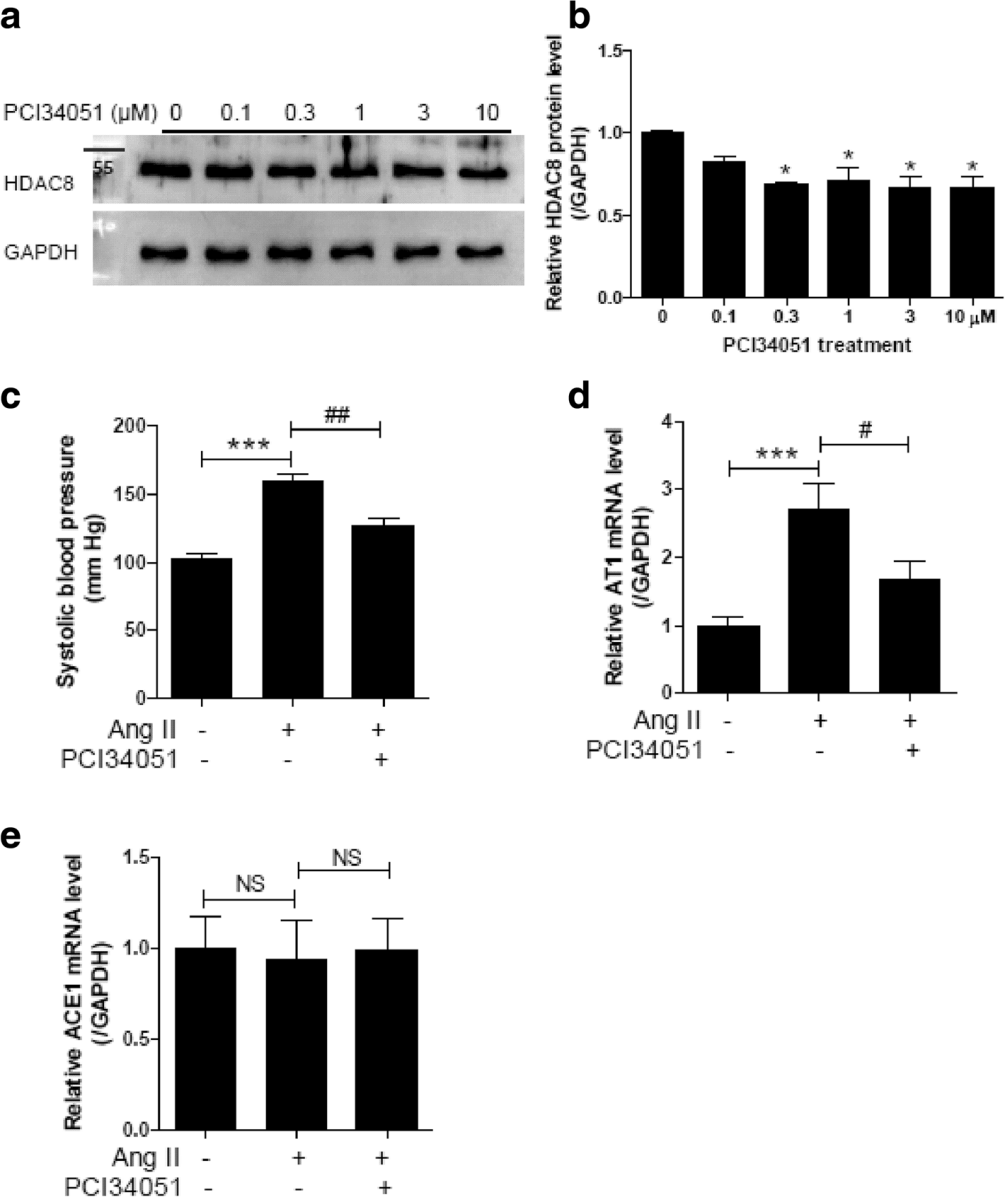
PCI34051 decreases blood pressure and AT1 mRNA expression in Ang II-infused mice. **a** Representative western blot images of HDAC8. VSMCs were treated with vehicle or PCI34051 at different concentrations for 24 h. **b** HDAC8 protein was quantified using densitometry. * *P* < 0.05 versus vehicle-treated VSMCs. **c** Systolic blood pressures in the experimental groups at 14th day after Ang II infusion. *** *P* < 0.001 versus sham group; ## *P*< 0.01 versus Ang II-infused group. **d** and **e** Aortic AT1 (**d**) and ACE1 (**e**) mRNA expression levels were normalized to GAPDH and relative amounts were calculated. Data are presented as mean ± SEM (n = 8 per group). *** *P* < 0.001 versus sham-treated group; # *P* < 0.05 versus Ang II-infused group; NS indicates not significant

PCI34051 is known to selectively inhibit HDAC8 [25, 26]. We confirmed that PCI34051 selectively inhibits HDAC8, with an IC50 of 0.02 μM. The IC50 for HDAC1 was 1.22 μM, while those for HDAC2 and HDAC3 were higher than 10 μM (Table 2). To determine whether PCI34051 affects HDAC8 expression in VSMCs, we performed western blot analysis. PCI34051 treatment was found to significantly reduce HDAC8 protein expression (Fig. 1a and b). To investigate the effect of the class I-selective inhibitor on hypertension, we tested PCI34051 in an Ang II-induced hypertensive mouse model. Systolic blood pressure significantly increased from 101.8 mmHg to 158.8 mmHg following two weeks of Ang II infusion. PCI34051 administration attenuated the increase in systolic blood pressure induced by Ang II (Fig. 1c). To address the potential antihypertensive mechanism of PCI34051, we studied the expression of AT1 and ACE1. As shown in Fig. 1d, AT1 mRNA levels were significantly higher in Ang II-infused mice than in sham-treated mice, and the increase was lower after PCI34051 treatment. However, no significant changes in ACE1 mRNA levels were found across the three groups (Fig. 1e).

**Table 2:** IC50 [μM] values for PCI34051

Compound	HDAC1	HDAC2	HDAC3	HDAC8
PCI34051	1.22	> 10	> 10	0.02

The in vitro inhibitory activity of PCI34051 against each HDAC isoform was determined using the HDAC Fluorogenic Assay Kit from BPS Bioscience. The IC50 values were determined using 0.0001, 0.0003, 0.01, 0.03, 0.1, 0.3, 1, and 3 μM of the inhibitor.

### PCI34051 reduces aortic wall thickness in Ang II-induced hypertensive mice

**Fig. 2:**
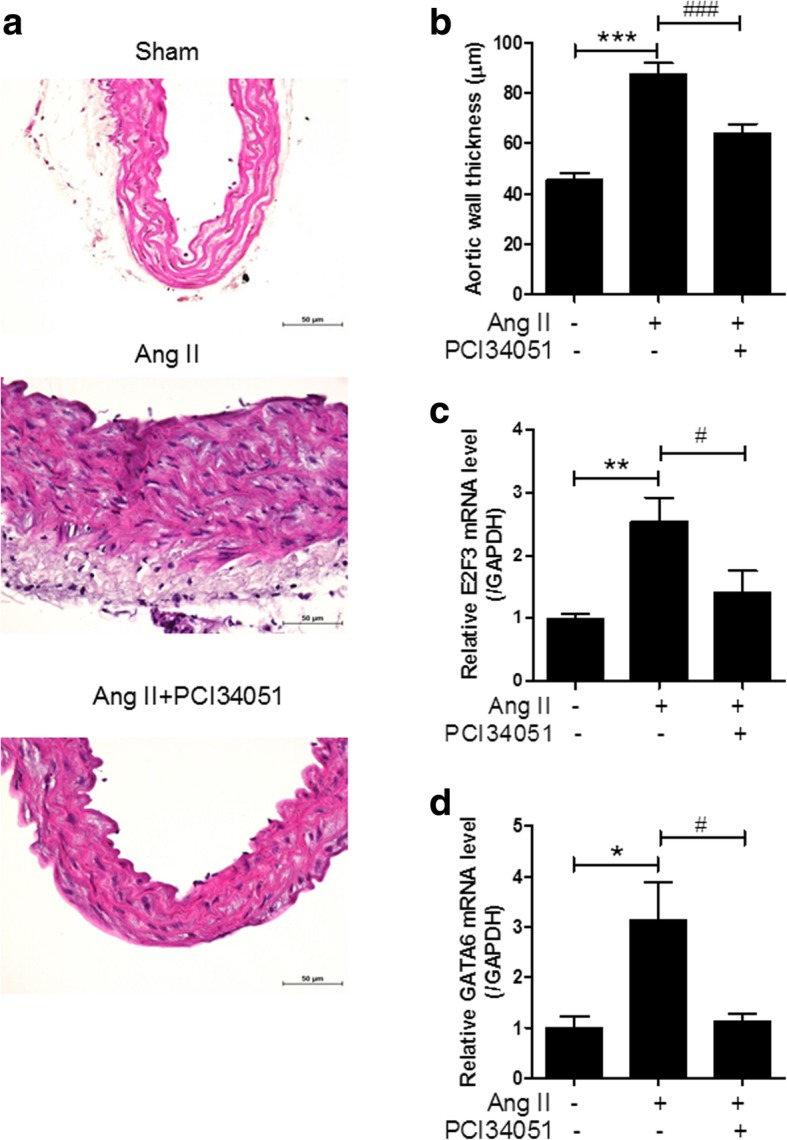
PCI34051 decreases vascular hypertrophy and expression of cell growth-related genes in Ang II-infused mice. **a** Representative images of H&E-stained aorta sections. Scale bar = 50 μm. **b** Aortic wall thickness was measured using NIS Elements Software (n = 8 per group). *** *P* < 0.001 versus sham group; ### *P* < 0.001 versus Ang II-infused group. E2F3 (**c**) and GATA6 (**d**) mRNA expression levels were normalized to GAPDH and relative amounts were calculated. Data are presented as mean ± SEM (n = 8 per group). * *P* < 0.05 and ** *P* < 0.01 versus sham-treated group; # *P* < 0.05 versus Ang II-infused group

It has been reported that Ang II induces the development of vascular hypertrophy as well as hypertension [27]. To identify whether PCI34051 regulates vascular hypertrophy, we measured aortic wall thickness after H&E staining. As shown in Fig. 2a, aortic wall thickness increased approximately 2-fold in Ang II-infused mice (87.2 μm) compared to that in the sham-treated group (45.4 μm). PCI34051 significantly decreased the enlarged aortic wall thickness in Ang II-infused mice (Fig. 2b). To explain the reduction of blood vessel thickness by PCI34051, RT-PCR was used to examine the changes in expression of genes related to cell proliferation. E2F3 and GATA6 mRNA expression levels significantly increased in the aortas of Ang II-infused mice compared to those of the sham-treated mice. These increases were attenuated by administration of PCI34051 (Fig. 2c and d).

### PCI34051 increases vascular relaxation in rat aortic rings and NO production in HUVECs

**Fig. 3:**
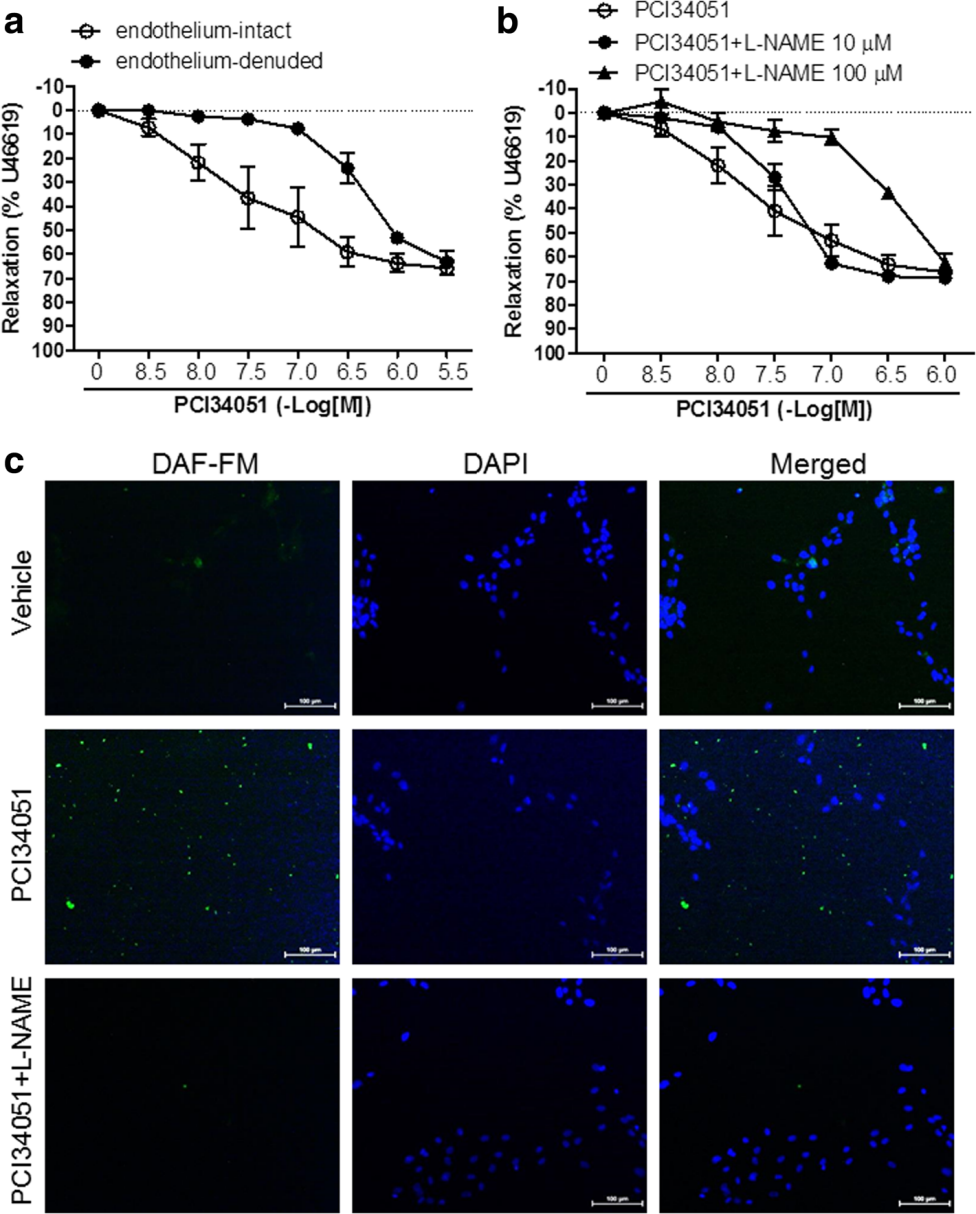
PCI34051 increases vascular relaxation in rat aortic rings and NO production in HUVECs. **a** Relaxation of aortic rings in response to PCI34051 was assessed in endothelium-intact and endothelium-denuded aortas. Relaxation is expressed as the percentage of the maximal contractile response to U46619. Data are presented as mean ± SEM (n = 4 per group). **b** Endothelium-intact rat aortic rings were pretreated with N^G^-nitro-L-arginine methyl ester (L- NAME, 10 μM or 100 μM) or vehicle for 30 min. When vascular contractions induced by U46619 (30 nM) reached a plateau, PCI34051 was added cumulatively to elicit relaxation. Data are presented as mean ± SEM (n = 4 per group). **c** HUVECs were treated with PCI34051 (1 μM) in the presence or absence of L-NAME (250 μM) for 24 h and then labeled with a fluorescent NO indicator, DAF-FM DA (2.5 μM), for 30 min. Three independent experiments were conducted and representative images are shown. Scale bar = 100 μm

A rat aortic ring test was performed to investigate whether the decrease in blood pressure after PCI34051 administration is associated with vascular tone. PCI34051 treatment induced vascular relaxation both in endothelium-intact and in endothelium-denuded aorta (Fig. 3a), although the effect was higher in endothelium-intact aorta.

We investigated whether vascular relaxation was related to the NO signaling system. We confirmed that pretreatment of the aortic rings with L-NAME (100 μM), a NOS inhibitor, prevented blood vessel relaxation by PCI34051 (Fig. 3b). Endothelial NO synthase (eNOS) synthesizes NO in vascular endothelium [28]. To explore whether blood vessel relaxation by PCI34051 is related to NO generation, we studied the effect of PCI34051 on NO production in HUVECs using a NO-sensitive fluorescent dye, DAF-FM. As shown by the increase in green fluorescence in Fig. 3c, PCI34051 treatment induced NO synthesis. Pretreatment with L-NAME reduced the amount of NO generated by PCI34051 in HUVECs.

### PCI34051 attenuates inflammation in Ang II-induced hypertensive mice

**Fig. 4:**
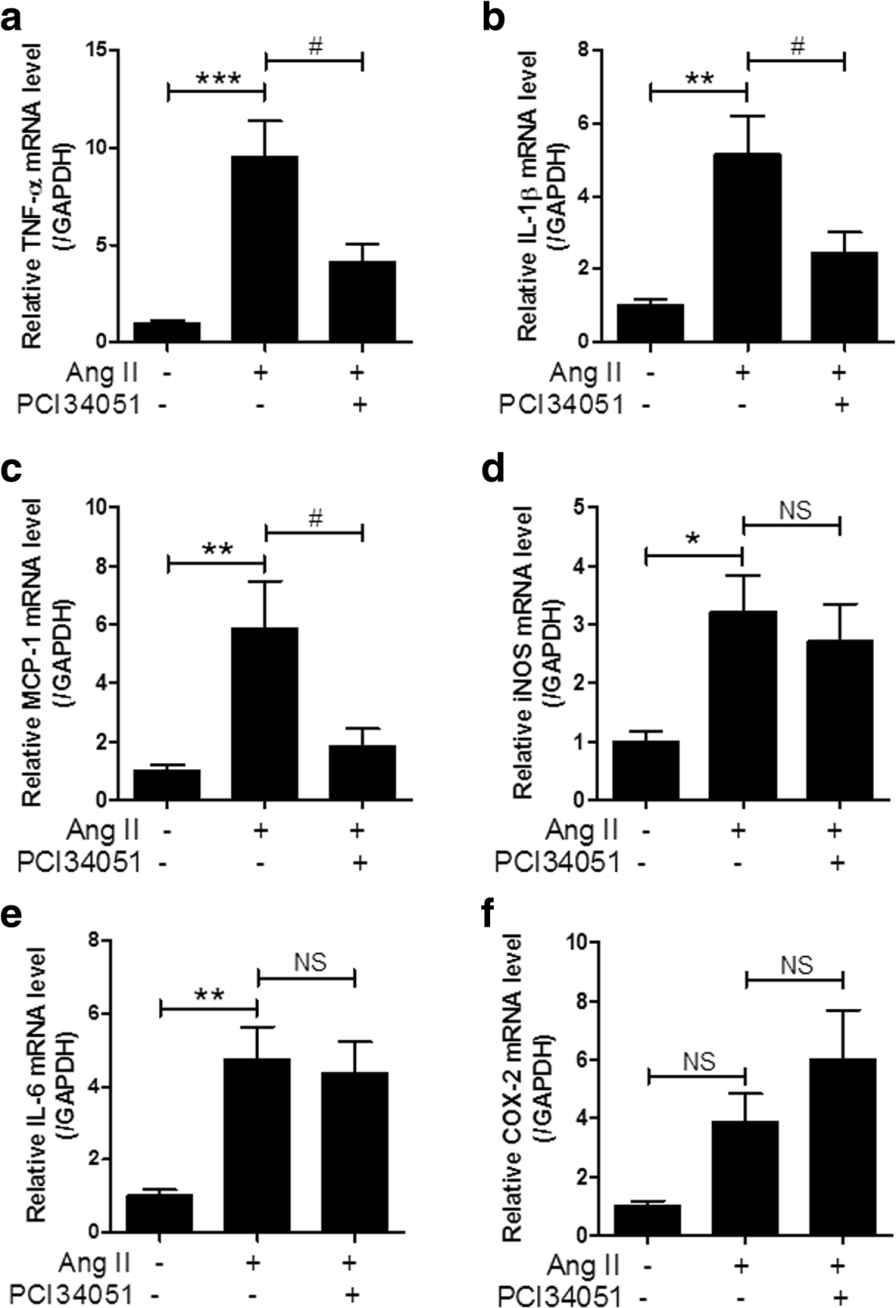
PCI34051 suppresses proinflammatory cytokines in aortic tissues of Ang II- infused mice TNF-α (**a**), IL-1β (**b**), MCP-1 (**c**), iNOS (**d**), IL-6 (**e**), and Cox-2 (**f**) mRNA expression in the aorta were normalized to GAPDH, and the relative expression was quantified. Data are presented as mean ± SEM (n = 8 per group). * *P* < 0.05, ** *P* < 0.01, and *** *P* < 0.001 versus sham-treated group; # *P* < 0.05 versus Ang II-infused group; NS indicates not significant

Many studies have reported that inflammatory responses participate in the pathophysiology of hypertension [5, 29, 30]. Tumor necrosis factor-α (TNF-α) regulates blood pressure in the kidneys during inflammatory conditions such as salt-sensitive hypertension [31, 32]. Interleukin-1 beta (IL-1β) levels are elevated in hypertension [33]. Monocyte chemoattractant protein-1 (MCP-1) is increased in Ang II-induced hypertension [34]. TNF-α, IL-1β, and MCP-1 are proinflammatory cytokines or chemokines. PCI34051 treatment significantly suppressed Ang II-induced TNF-α, IL-1β, and MCP-1 mRNA expression in the aorta (Fig. 4a-c). High NO levels are detrimental to the cardiovascular system and are associated with inducible NOS (iNOS) expression [35]. IL-6 expression is higher in hypertensive animal models and hypertensive humans than in normotensives [36]. The expression of iNOS and IL-6 mRNA significantly increased in the aorta tissues of Ang II-infused mice but was not affected by PCI34051 treatment (Fig. 4d and e). Cyclooxygenase-2 (COX-2) is enhanced in vascular smooth muscle cells in response to an Ang II stimulus [37]. However, there was no significant difference in the expression of COX-2 between groups (Fig. 4f).

### PCI34051 reduces adhesion molecules in Ang II-induced hypertensive mice

**Fig. 5:**
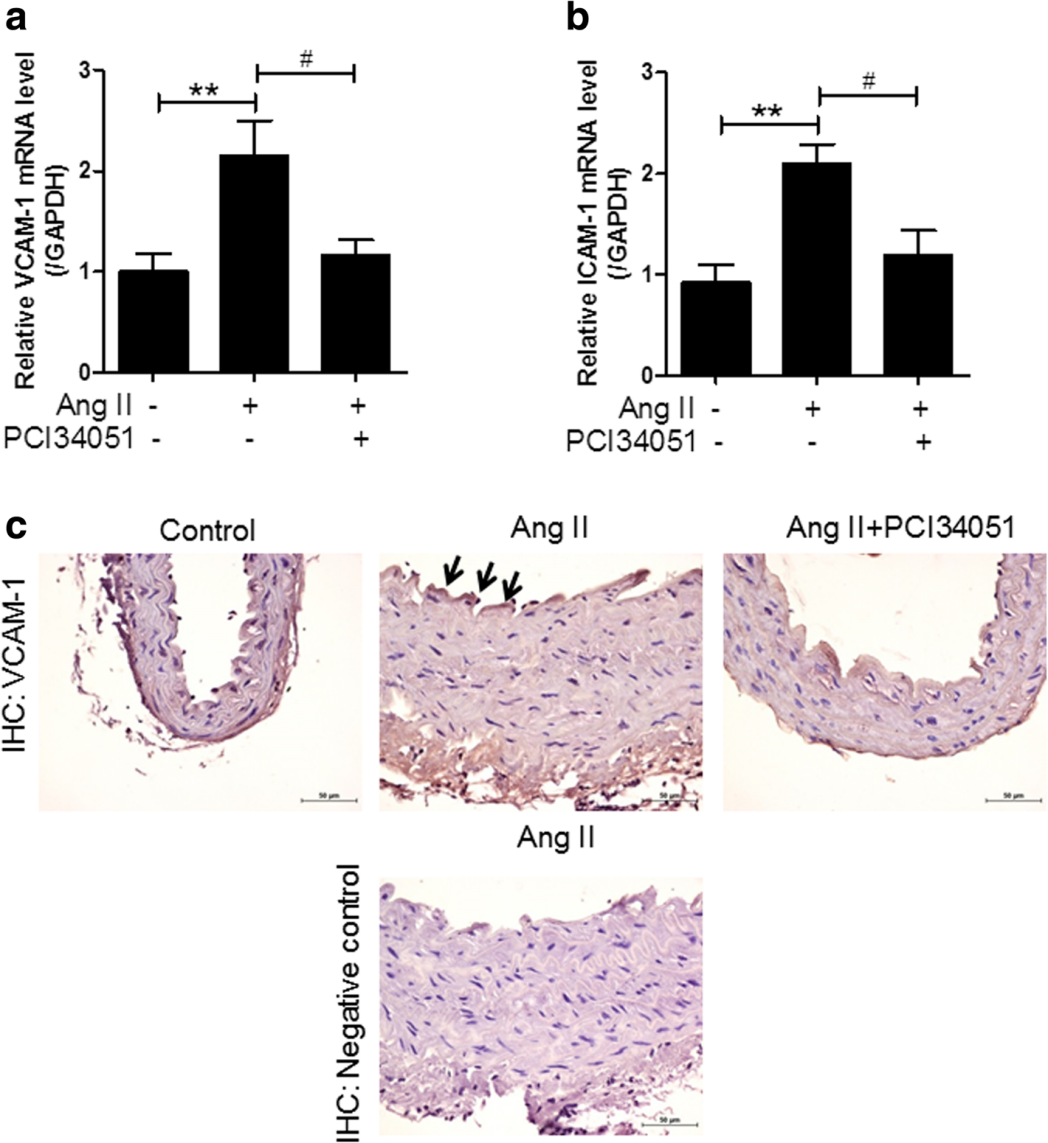
PCI34051 inhibits the expression of adhesion molecules in aortic tissues of Ang II-infused mice VCAM-1 (**a**) and ICAM-1 (**b**) aortic mRNA expression levels were normalized to GAPDH and relative amounts were calculated. Data are presented as mean ± SEM (n = 8 per group). ** *P* < 0.01 versus sham-treated group; # *P* < 0.05 versus Ang II-infused group. **c** VCAM-1 expression was assessed by immunohistochemistry. Arrows indicate the expression of VCAM-1 in the aorta (brown). Scale bar = 50 μm. The lower Ang II-infused group image shows a negative control.

Adhesion molecules mediate the interactions between blood cells and endothelial cells under pathological conditions [38]. Adhesion molecules, including ICAM-1, VCAM-1, and E- selectin, are located on the surface of endothelial cells and are highly increased in the blood in response to pathological conditions [39, 40]. Hypertensive patients have been reported to have higher circulating levels of soluble ICAM-1 (sICAM-1) and VCAM-1 (sVCAM-1) than normotensive people [11]. The mRNA levels of VCAM-1 and ICAM-1 were higher in the aortas of the Ang II-infused mice than in those of the control group and were significantly decreased by PCI34051 treatment (Fig. 5a and b). Immunohistochemistry revealed that VCAM-1 was highly expressed in the endothelium of Ang II-treated aortas compared to those of the controls (Fig. 5c). After treatment with PCI34051, VCAM-1 expression was attenuated.

### PCI34051 does not affect NADPH oxidase (Nox) in Ang II-induced hypertensive mice

**Fig. 6:**
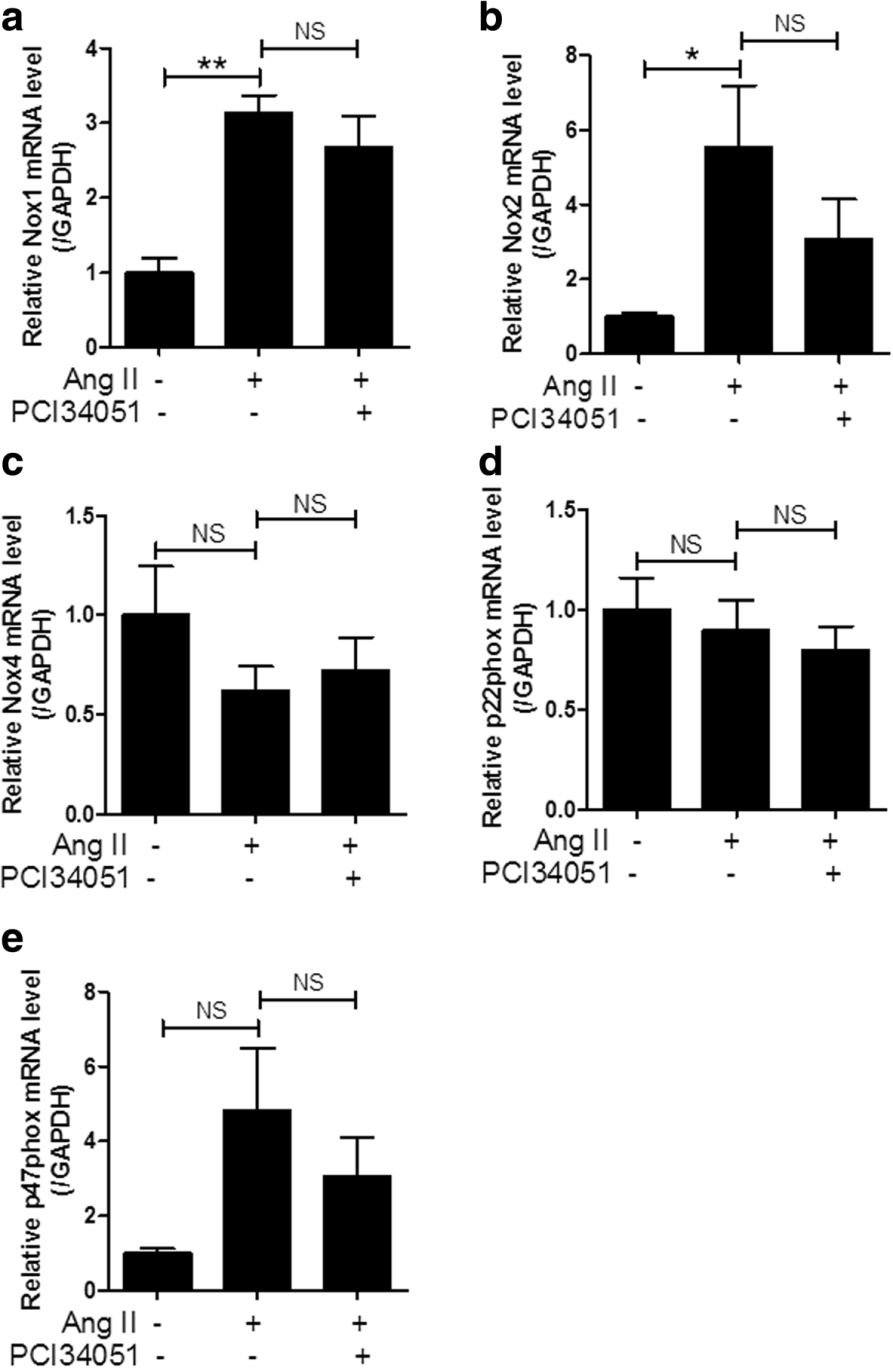
PCI34051 does not affect Nox isoforms or Nox subunits in Ang II-infused mice. Aortic mRNA expression levels of Nox1 (**a**), Nox2 (**b**), and Nox4 (**c**), and Nox subunits p22phox (**d**) and p47phox (**e**), were normalized to GAPDH and relative amounts were calculated. Data are presented as mean ± SEM (n = 8 per group). * *P* < 0.05 and ** *P* < 0.01 versus sham-treated group; NS indicates not significant

Ang II is associated with oxidative stress in hypertension. Nicotinamide adenine dinucleotide phosphate (NADPH) oxidases (Nox) generate reactive oxygen species (ROS), leading to vascular damage [41]. The expression of Nox1 and Nox2 mRNA were significantly increased in the Ang II-infused group compared to the sham-treated group, but this was not attenuated by PCI34051 administration (Fig. 6a and b). The p22phox is an essential component of the membrane-associated enzyme phagocyte Nox and the p47phox is a regulatory subunit of Nox. However, Nox4 and p22phox mRNA expression was not increased in the Ang II-infused group (Fig. 6c and d). Although not significant, p47phox mRNA showed an increasing trend after Ang II administration, but this was not affected by PCI34051 administration (Fig. 6e).

## Discussion

In the present study, we demonstrated that PCI34051, an HDAC8-selective inhibitor, lowered systolic blood pressure, reduced aortic wall thickness, increased vascular relaxation, and inhibited inflammation in a mouse model of Ang II-induced hypertension.

Although there are reports that HDAC inhibitors lower blood pressure [21] in various diseases including essential hypertension, high-fat diet-induced hypertension [42], and Cushing’s syndrome [43], in many circumstances we do not know which isoform of HDAC is involved. Initially, in a cell-free system, we observed that PCI34051 selectively inhibits HDAC8 enzyme activity, but not the activities of HDAC1, HDAC2, or HDAC3.

Ang II causes hypertension through effects on the AT1 receptor, leading to vascular contraction [44]. It is well known that ARBs act by selectively blocking the binding of Ang II to the AT1 receptor [45]. In the present study, we observed that PCI34051 administration significantly decreased systolic blood pressure in Ang II-infused mice by decreasing AT1 mRNA expression. However, blood pressure was not fully returned to control levels by PCI34051 administration, implying that other HDACs may be involved, in addition to HDAC8.

There have been reports that drugs such as calcium-calmodulin-dependent kinase II inhibitor (KN-93) and HDAC inhibitor (MC1568) are effective in reducing Ang II-induced vascular hypertrophy [24, 46]. In the present study, the HDAC8-selective inhibitor, PCI34051, was also effective in partially reducing the vascular hypertrophy induced by Ang II. In our previous research [24], GATA6 transcription factor directly increased the size of vascular smooth muscle cells. E2F3 is well known as a transcription factor that regulates cell proliferation [47]. Our results show that the inhibitory effect of PCI34051 on vascular hypertrophy may be due to its inhibition of GATA6 and E2F3 expression, as a decrease in the mRNA expression of these transcription factors was seen in the Ang II-infused mice after PCI34051 administration.

The most interesting finding is that PCI34051 relaxes blood vessels in rat aortic rings. The blood vessel relaxation by PCI34051 is particularly dependent on endothelial cells and is effected through the NO signaling system. Indeed, we demonstrated that PCI34051 treatment induced NO production in HUVECs and that this could be blocked by a NOS inhibitor.

This result suggests that HDAC8 activity is closely related to vascular contraction and relaxation. In contrast to our results, Lee et al reported that a pan-HDAC inhibitor, CG200745, had little effect on vascular relaxation in DOCA-induced hypertension [48]. However, in agreement with our results, SAHA, a pan-HDAC inhibitor, or trichostatin A (TSA) caused a dose-dependent relaxation of the phenylephrine-induced vascular contraction of mouse aortas or the Ang II-induced contraction of rat aortas [49, 50]. Long-term treatment with TSA inhibited Ang II-induced contraction in spontaneously hypotensive rats [51]. Some researchers have used pan-HDAC inhibitors, so we cannot be sure which HDACs have an effect on vascular relaxation.

Ang II induces vascular inflammation by inducing proinflammatory cytokines [52]. The HDAC8-selective inhibitor decreased the expression of the proinflammatory cytokines, TNFα, IL-1β, and MCP-1, and suppressed inflammation, suggesting that these two effects may be linked. Adhesion molecules are also implicated in inflammation. ICAM-1 and VCAM-1 mediate adhesion of leukocytes to the endothelium. sICAM-1, sVCAM-1, P-selectin, and E- selectin levels have been seen to be higher in hypertensive patients [53]. In addition, the expression of VCAM-1 was increased in the aortas and mesenteric resistance arteries of Ang II-induced hypertensive mice [54]. Our current study shows that treatment with an HDAC8- 18 selective inhibitor suppresses the increased expression of VCAM-1 and ICAM-1 in Ang II- induced hypertension. Furthermore, immunohistochemistry demonstrated that VCAM-1 protein was highly expressed in the endothelium of aorta tissues in Ang II-infused mice compared to that in control mice. Our findings suggest that HDAC8 activity may well be responsible for vascular inflammation.

Ang II regulates ROS through upregulation of Nox [41]. Angiotensin II-induced ROS can cause vasoconstriction, inflammation, and vascular remodeling through activation of multiple pathways. In our study, Ang II increased the aortic expression of Nox1 and Nox2 mRNA but did not affect the expression of Nox4. The HDAC8-selective inhibitor did not decrease Nox1 and Nox2 expression. This result implies that HDACs other than HDAC8 modulate Nox expression. Cardiac Nox1, Nox2, and Nox4 mRNA levels significantly increased in SHR hypertensive rats compared to that in Wistar Kyoto (WKY) control rats [55]. Gallic acid, which inhibits HDAC5, HDAC7, HDAC8, and HDAC9, ameliorated Nox2 mRNA and protein levels in SHR [56]. Manea et al. reported that SAHA decreased the aortic expression of Nox1, Nox2, and Nox4 in diabetic mice by regulation of HDAC1 and HDAC2 [57]. In contrast, increased expression of HDAC3, HDAC4, and HDAC5 is associated with induction of Nox2 and Nox4 in pulmonary arterial hypertension, which was decreased by the HDAC inhibitor, valproic acid [58]. The results of the above study show that the expression of Nox varies depending on the type of disease; for example, arterial hypertension, pulmonary hypertension, or diabetes. The pan-HDAC inhibitor was more effective in reducing the expression of Nox than an HDAC isoform-specific inhibitor.

## Conclusions

We demonstrated that an HDAC8-selective inhibitor lowered blood pressure, inhibited vascular hypertrophy and inflammation, and relaxed blood vessels in an Ang II-induced hypertension model. The HDAC8-selective inhibitor contributed to blood pressure reduction by inhibiting a component of the RAS or regulating NO signaling pathways. We suggest that HDAC8 may be a therapeutic target for hypertension.

## Abbreviations

Ang II: Angiotensin II
ARB: Angiotensin II receptor blocker
AT1: Angiotensin II receptor type I
COX-2: Cyclooxygenase-2
DAF-FM DA: 4-amino-5-methylamino-2′,7′-difluorofluorescein diacetate
DOCA: Deoxycorticosterone acetate
EBM: Endothelial cell basal medium
H&E: Hematoxylin and eosin
HDACs: Histone deacetylases
HUVECs: Human umbilical vascular endothelial cells
IC50: Half-maximal inhibitory concentration
ICAM-1: Intercellular adhesion molecule-1
IL-1β: Interleukin-1 beta iNOS: inducible nitric oxide synthase
L-NAME: NG-nitro-L-arginine methyl ester
MCP-1: Monocyte chemoattractant protein-1
NADPH: Nicotinamide adenine dinucleotide phosphate
NO: Nitric oxide
Nox: NADPH oxidase
PECAM: Platelet endothelial cell adhesion molecule
sVCAM-1: soluble VCAM-1
TNF-α: Tumor necrosis factor-α
VCAM-1: Vascular cell adhesion molecule-1
